# *Drosophila* Heart as a Model for Cardiac Development and Diseases

**DOI:** 10.3390/cells10113078

**Published:** 2021-11-08

**Authors:** Anissa Souidi, Krzysztof Jagla

**Affiliations:** Genetics Reproduction and Development Institute (iGReD), INSERM U1103, CNRS UMR6293, University of Clermont Auvergne, 28 Place Henri-Dunant, 63000 Clermont-Ferrand, France; anissa.souidi@uca.fr

**Keywords:** heart, cardiogenesis, *Drosophila melanogaster*, conduction defects, arrhythmia, congenital cardiomyopathy, myotonic dystrophy type 1

## Abstract

The *Drosophila* heart, also referred to as the dorsal vessel, pumps the insect blood, the hemolymph. The bilateral heart primordia develop from the most dorsally located mesodermal cells, migrate coordinately, and fuse to form the cardiac tube. Though much simpler, the fruit fly heart displays several developmental and functional similarities to the vertebrate heart and, as we discuss here, represents an attractive model system for dissecting mechanisms of cardiac aging and heart failure and identifying genes causing congenital heart diseases. Fast imaging technologies allow for the characterization of heartbeat parameters in the adult fly and there is growing evidence that cardiac dysfunction in human diseases could be reproduced and analyzed in *Drosophila*, as discussed here for heart defects associated with the myotonic dystrophy type 1. Overall, the power of genetics and unsuspected conservation of genes and pathways puts *Drosophila* at the heart of fundamental and applied cardiac research.

## 1. Introduction

With its short life cycle (10 days at 25 °C), high offspring numbers (2000 eggs per female), low maintenance costs, and conservation of genes and cellular pathways, *Drosophila* is an outstanding model for genetic studies.

Developed over the years, molecular and genetic tools, such as the GAL4/UAS conditional gene expression system [1] and numerous collections of mutants, RNAi knockdown and transgenic lines along with CRISPR/Cas9 technology tools [2,3,4], and recently implemented model organism resources [5], greatly facilitate the analysis of gene functions.

The sequencing and annotation of the *Drosophila* genome [6] revealed that genes involved in the development of several organs, including the heart, were highly conserved and less redundant than in vertebrates. Remarkably, 77% of human disease genes have *Drosophila* counterparts, among which 26 were identified as associated with cardiovascular diseases [7]. Such conservation of genes, signaling pathways, and cellular processes, make the *Drosophila* cardiovascular system an amenable genetic model to study cardiac development, function, and diseases [8].

In this review, we discuss the mechanisms of *Drosophila* heart development and how this simple model is applied to the study of congenital heart diseases. We also discuss how the model of the adult fly heart helps in dissecting the mechanisms of human cardiac aging, heart failure, and those underlying cardiac defects in myotonic dystrophy type 1 (DM1)—one of the most frequent myopathies in humans.

## 2. The *Drosophila* Heart

The *Drosophila* heart is derived from the dorsal-most mesoderm and consists of two major cell types: the cardioblasts (CBs), which form the cardiac tube and differentiate into contractile cardiomyocytes, and the pericardial cells (PCs), which are irregularly arranged on both sides of the heart and perform hemolymph filtration [9,10]. The dorsal vessel has an anterior–posterior polarity with an anteriorly located “aorta” and the posterior “heart proper” [9]. A pair of specialized CBs that form a cardiovascular valve separate the heart and the aorta. The hemolymph, which supplies the organs with nutrients, enters the heart proper via inflow tracts termed ostia, and is ejected via an outflow tract located at the tip of the aorta [11].

### 2.1. Cardiac Development in Embryos

**Figure 1:**
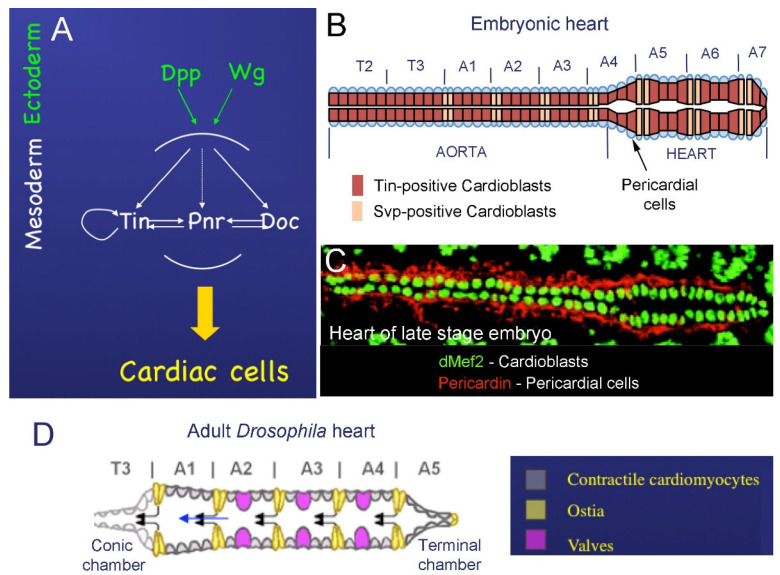
The embryonic and adult fly heart. (**A**) Cardiogenic pathway: ectodermal signals and cardiac master genes required for the specification of cardiac cells. (**B**) Scheme of the embryonic cardiac tube subdivided on the anterior aorta and the enlarged posteriorly located heart proper. Color code represents the Tin- and Svp-positive subpopulations of cardioblasts. (**C**) A dorsal view of the cardiac tube in a late-stage embryo. Nuclei of cardioblasts are revealed by Dmef2 (green) whereas surrounding pericadial cells are marked by pericardin (red). (**D**) Scheme of the adult heart after remodeling. Different types of cardiac cells within the heart segments are represented by grey, yellow, and violet colors. Conical chamber forms the anterior and terminal chamber at the posterior heart end. Arrows indicate the direction of hemolymph flow.

At the embryonic blastoderm stage, maternal signaling events initiate the expression of the master mesodermal transcription factor Twist and specify the primordial cells of the mesoderm. These cells invaginate along the ventral midline into the interior of the embryo and then by embryonic stage 9 spread laterally and dorsally into a monolayer of cells opposed to the ectoderm [10]. The specification of the cardiac mesoderm and other mesoderm-derived tissue types begins at stage 10 under the influence of two growth factors, Decepentaplegic (Dpp) and Wingless (Wg), expressed in the ectoderm (Figure 1A); Wg in continuous stripes [10,12], and Dpp in a broad dorsal band [12]. *Wg* and *Dpp* collectively activate *tinman* (*tin*), which encodes for the mesodermal homeodomain transcription factor and acts to determine cardiac and visceral mesodermal fates. Soon after, at early embryonic stage 11, *Dpp* and *Wg* induce the expression of GATA factor *Pannier (Pnr)* in the presumptive cardiac mesoderm, and in parallel ensure the restricted *Tin* expression, which then acts as a cardiac master gene [12,13].

The maintenance and proper restriction of *Tin* expression and positive feedback loop between Pnr, Tin, and another cardiac transcription factor, Doc (Figure 1A), are essential for cardiomyocyte differentiation.

From stage 12 to stage 15, the two rows of *Tin*-positive cardioblasts align and migrate dorsally toward each other to ultimately form the cardiac tube (schematized in Figure 1B). Their migration is initially supported by the overlying dorsal ectoderm, but final movements and the CB matching are ectoderm independent [14]. The heart spans over two thoracic and seven abdominal segments, and cardiac cells (CBs and PCs) are arranged in repeated segmental units (Figure 1B). In late-stage embryos, each segment has four pairs of CBs that express *Tin* and two pairs of *Svp*-positive CBs [15] surrounded by *pericardin*-expressing PCs (Figure 1C). Two out of four *Tin*-positive CBs also express the *ladybird* (*lb*) homeobox genes acting as cell identity genes [16].

In addition, all CBs express two other conserved transcription factors, *Myocyte enhancer factor 2* (*Mef2*) (see Figure 1C) and *Hand* [17].

### 2.2. Development of the Adult Fly Heart—Hox-Dependent Remodeling of the Cardiac Tube

As previously described, the cardiac tube is divided into the “heart proper” and the anteriorly located “aorta”. In the developing embryo, the anteroposterior polarity determination of the dorsal vessel is controlled by homeotic Hox genes of the *Bithorax complex* (*ultrabithorax* (*Ubx*), *abdominal-A (abdA)*, *abdominal-B (abdB)* and *Antennapedia complex* (*Antp*) [18]. In the embryo, *Ubx* is expressed in different portions of the aorta and determines the identity of aorta CBs, while *abdA* is expressed in cardiac segments A5 to A7 and is required for the proper specification and differentiation of the heart [19,20]. *abdB* expression correlates with the posterior terminus of the heart. Experiments of gain of function and loss of function of *Hox* genes demonstrated that the specific expression of *Hox* genes along the cardiac tube was necessary for its anteroposterior polarity and for proper cardiomyocyte differentiation [19,20].

The adult fruit fly heart is formed during metamorphosis by the reprogramming of differentiated and functional larval cardiomyocytes, without cell proliferation. Heart remodeling involves the regulation of *Hox* gene expression and function driven by the steroid hormone ecdysone [18]. Ecdysone modulates *Ubx* activity in *svp*-expressing cells and promotes adult ostia development. The modulation of *Ubx* expression induces the remodeling of larval aorta myocytes into a functional adult heart, whereas *abdA* mediates the transformation of the surviving larval contractile heart myocytes into the adult terminal chamber [18].

The adult heart is remodeled from the larval dorsal vessel in the following way: (1) The larval posterior aorta (segments A1 to A4) undergoes remodeling into the adult heart (schematized in Figure 1D), while the larval heart (abdominal segments A5–A7) is largely histolyzed by programmed cell death. (2) In A1–A4, the *Tin*-expressing myocytes acquire contractile activity and *Ubx* transforms presumptive ostial cells (*Svp*-positive cells) from the larval aorta into functional ostia, so that there are four pairs of ostia located in the adult abdomen [18,21,22] (Figure 1D). (3) Three anterior pairs of alary muscles trans-differentiate into a layer of striated muscle, called ventral longitudinal muscle (VLM). The VLM is formed on the ventral surface of the adult heart and is separated from the heart by a basal lamina [22,23,24]. (4) Three valves are formed in segments A2, A3 and A4 [23,24] (Figure 1F). (5) Cardiomyocytes in segment A5 undergo a trans-differentiation process; they lose contractile activity and gain specific innervations to form the terminal chamber [18]. (6) The number of pericardial cells surrounding the adult heart is reduced, most probably via programmed cell death [24].

## 3. Vertebrate Heart and Its Similarity with the *Drosophila* Cardiac Tube

The heart is the first functional organ in vertebrate embryos, including in humans [25]. It is derived from the anterior portion of the lateral plate mesoderm and is initially formed as a simple contractile vessel called the “heart tube”, oriented along the ventral midline of the foregut, and then transformed by elongation, looping, and morphological remodeling into a multi-chambered organ that propels oxygenated blood at high pressure via blood vessels [26].

The cardiac morphogenesis in vertebrates takes place in four steps. (1) Determination and specification of cardiomyocytes. (2) Formation of the cardiac tube. (3) Looping and chamber formation. (4) Formation of valves and septation.

The first step takes place during gastrulation. It begins by the specification of cardiac progenitor cells in the anterior lateral plate mesoderm and the formation of the cardiac crescent. The commitment of mesodermal precursors to cardiac progenitor cells involves signaling from the adjacent endoderm and ectoderm, including Wg, FGF, and TGF-β pathways [25]. After specification, the cardiac progenitor cells migrate through the node and primitive streak to develop specific heart fields, which form the cardiac crescent. The bilateral heart fields of the cardiac crescent then start expressing cardiac-specific transcription factors such as Nkx2.5 and Isl1. They migrate under the forces generated by the closure of the anterior intestinal portal, and fuse anteriorly at the ventral midline to form a primitive cardiac tube. At this time, around embryonic day 22 in humans, the primitive cardiac tube begins to contract and pump blood, driven by pacemaker activity in the venous pole [25,26].

The next stage of heart morphogenesis is “looping”, whereby the primitive cardiac tube is transformed into a helically coiled heart loop. During this step, the cardiac tube bends to the right and initiates looping by a rapid expansion and elongation followed by a folding and twisting process. Elongation begins by the addition of newly differentiated cardiomyocytes to the arterial and venous poles. These cells originate from the distal mesoderm, proliferate rapidly, migrate, and enter the heart tube. This cell population is called the “anterior heart field” (AHF) or “second heart field” (SHF) as opposed to the “first heart field” (FHF) from which the initial heart tube was formed.

Cells of the FHF contribute to the left ventricular wall, part of the ventricular septum, and a portion of the atria, whereas the SHF cells mainly form the outflow tract, part of the atria, and also contribute to the right ventricle. After cardiac looping, the primary heart tube undergoes division into four well-defined chambers. This is followed by septation and remodeling of the heart, which becomes connected to partitioned systemic and pulmonary circulations [27].

Despite obvious morphological differences, there are many similarities between the *Drosophila* cardiac tube and the vertebrate heart. Developmentally, *Drosophila* and vertebrates have cardiac progenitors of similar origin. In both cases, the heart precursors are derived from the bilateral rows of mesodermal cells that migrate to the midline and fuse together to form a beating linear cardiac tube. This simple cardiac tube acquires anterior–posterior polarity and pumps the hemolymph in *Drosophila* and blood in vertebrates from the lower/posterior part to the upper/anterior part [8,28].

Remarkably, the cardiac progenitor fate specification in both systems requires the same inductive Dpp/TGF-β and Wg/Wnt signals emanating from adjacent ectodermal and/or endodermal cells and activating key cardiac transcription factors such as Tin/Nkx2.5, Dmef2/Mef2C, Panier (Pnr)/GATA, and dHand/HAND (see Table 1) [8,16,29].

Unlike the vertebrate heart, the cardiac tube of *Drosophila* does not undergo looping and remains a simple linear tube. However, its final morphogenesis and formation of the cardiac outflow tract (OFT) involves, as in vertebrates, a population of non-mesodermal cells called heart-anchoring cells (HANC) [11,30], which are to some extent analogous to vertebrate cardiac neural crest cells (cNCCs) [31]. Moreover, in both *Drosophila* and vertebrates, formation of the OFT requires cells originating from the pharyngeal mesoderm. These are progenitors of two cardiac outflow muscles (COMs) [11,30] in fruit fly heart and the OFT-building secondary heart field (SHF) cells in vertebrates. Functionally, the vertebrate heart is part of a closed circulatory system. By pumping the oxygen-transporting blood cells, it ensures gas exchange and so is critical for viability. By contrast, the *Drosophila* heart pumps the hemolymph in an open circulatory system. It is required for transporting nutrients and signaling entities but not oxygen (distributed by the tracheal system), and so is not essential for viability [8,28,32]. In this light, *Drosophila* heart represents an attractive system for modeling human cardiac diseases, causing lethality in vertebrate models.

**Table 1:** Genes involved in cardiac development conserved between *Drosophila* and vertebrates.

Fly Gene	Expression	Mutants	Vertebrate Ortholog	Expression	Mutants
*Tinman*	Expressed early during development uniformly in mesoderm After gastrulation: restricted to the dorsal portion of the mesoderm Later: transiently in visceral mesoderm and permanently in heart (in cardiac and pericardial cells) [8,28]	Lack of visceral and cardiac mesoderm. The somatic mesoderm shows moderate patterning defects later in development [28,33]	*Nkx2–5*	Initially expressed in the bilateral cardiac progenitors of the anterior lateral plate mesoderm and in part of the pharyngeal endoderm	Lack of ventricular-specific myosin light-chain gene expression Heart tube fails to undergo normal looping [34]
*Dmef2*	Expressed myogenic precursor lineages and their descendants [8]	Heart differentiation affected [35]	*Mef2*	Expressed in cardiac, skeletal, and smooth muscle precursor lineages [8]	Defects in heart looping and Hand2 downregulation [8]
*HAND*	Heart, lymph glands, circular visceral musculature, and a subset of CNS cells	In embryos: lack of lymph glands. In adult: disorganized myofibrillar structure, reduced systolic and diastolic diameter, abnormal heartbeat contractions, midguts highly deformed, and premature lethality [36]	*Hand2/3*	Heart neural crest derivatives [37]	Aortic sac defects Heart looping defects [37]
*Dpp*	Dorsal ectoderm	Lack of heart and visceral mesoderm [8]	*BMPs*	Expressed in endoderm and ectoderm	Affected heart development Down regulation of Nkx2–5 [38]
*Wg*	Ectoderm—adjacent to cardiac mesoderm [39]	Loss of repeated clusters of even-skipped expressing cells in mesoderm [39] Loss of heart precursors [10]	*Wnt*	Wnt5a and Wnt11 expressed in second heart field [40]	Defective right ventricle development [41]

## 4. Identifying Cardiac Aging Genes and Modeling Human Heart Diseases in *Drosophila*

Besides the conservation of gene regulatory pathways, there is growing evidence that mutations in genes involved in cardiac diseases cause fly heart phenotypes similar to those observed clinically in humans [7]. *Drosophila* is used as a model organism for studying physiological, genetic, and epigenetic bases of cardiac aging [42]. There is also growing interest in using *Drosophila* to dissect molecular mechanisms of cardiac disease- and aging-associated heart failure [43].

In this part of the review, we focus on the application of *Drosophila* to identifying new genes implicated in cardiac aging, heart failure, and in congenital heart diseases, and as an example, discuss how the fly model helps us gain an understanding of cardiac defects associated with myotonic dystrophy type 1 (DM1), a toxic transcript repeat disease. Overall, this makes *Drosophila* an attractive model system for dissecting mechanisms of human cardiovascular diseases.

### 4.1. Studying Cardiac Aging and Heart Failure in Drosophila

Similar to mammals, flies present age-related decline in cardiac performance with a lower heart rate and increased fibrillation [44,45] particularly evident in stress conditions. To identify the changes in gene expression that underlie cardiac aging, Cannon and co-workers [46] analysed cardiac transcriptomes of young and old flies and compared the genes differentially expressed in the old fly heart with genes known to be involved in cardiac aging in mammals. They found the upregulation of extracellular matrix (ECM) genes, and genes involved in DNA replication and repair. In contrast, genes involved in ATP synthesis and β-oxidation, as well as in carbohydrate metabolism, were downregulated [46]. Among upregulated-with-age ECM genes are *Matrix Metalloprotease 1* and *2* (*Mmp1* and *Mmp2*), *neprilysin 2* (*Nep2*), and *TweedleF* (*TwdlF*) [46], as well as cardiac collagen-IV (*Viking*) and *pericardin* (encoding a collagen IV-like protein) [42].

*In silico* analysis of genes upregulated with age in the fly heart, performed by Cannon et al. [46], identified a significant enrichment of binding sites for several micro-RNAs including *miR-1*, an evolutionarily conserved muscle- and heart-specific miRNA. The level of *miR-1* was found to be significantly reduced in aging *Drosophila* hearts concomitant with the overexpression of the zinc-finger transcription factor *odd*, a critical transcriptional regulator in patterning the body plan of the *Drosophila* embryo. These results suggest that *miR-1* and *odd* could play a role in cardiac aging by regulating the expression of their targets. Among them, *Matrix metalloprotease 1* (*Mmp1*) was found to be upregulated in old fly hearts. The overexpression of *Mmp1* in heart tissue leads to a reduction in fractional shortening, suggesting its implication in cardiac aging [46].

Experiments performed by Occor and co-workers [47] also revealed an elevated incidence of cardiac dysfunction in aging flies. In this study, authors identified a decrease in the efficacy of cardiac relaxation/repolarization due to the affected voltage-gated potassium channel (*KCNQ*) function. It was found that young *KCNQ1* mutant flies exhibit several cardiac abnormalities in old wild-type flies, including slower heart rate and an increased susceptibility to pacing-induced cardiac dysfunction (heart failure), thus suggesting the involvement of *KCNQ* in cardiac aging [47].

Heart failure corresponding to a decline in the capacity to pump blood is a common consequence of various heart diseases, and in particular dilated and hypertrophic cardiomyopathies. Deregulations of genes and pathways involved in cardiac aging such as Insulin Growth Factor (IGF) and the Target of Rapamycin (TOR) pathway [48,49], and genes encoding ion channels such as *Ca/Calmodulin-dependent protein Kinase II* (*CaMKII*) [50] have been previously shown to play a role in heart failure [43,51]. Heart failure is also associated with aberrant cytoskeletal remodelling. For example, missense mutations in vinculin encoding integrin-like protein are associated with an increased incidence of heart failure in humans, whereas cardiac overexpression of vinculin in flies positively influences heart performance and increases lifespan [52,53]. A greater susceptibility to heart failure has also been observed in mutants of the chromatin remodelling via *Carbon Catabolite Repression 4* (*CCR4*)–*Negative on TATA-less* (*Not*) (*CCR4–Not*) complex (Not3), leading to cardiac contractility defects in mice and dilated cardiomyopathy in flies [54,55].

### 4.2. Identifying Genes Involved in Congenital Heart Defects

By definition, congenital heart defects (CHDs) are heterogeneous abnormalities of the heart or the great vessels (aorta, pulmonary artery, pulmonary veins) at the time of birth. CHDs are the most common birth defects, occurring in about 0.8% of all newborn infants [56]. However, despite extensive molecular and genetic analyses using chromosomal microarrays, whole-exome sequencing, and next-generation sequencing technologies [57], the genes causal for about 75% of CHD cases remain unknown [58].

To fill this gap, several animal models have been studied, including zebrafish, frog, chick, mice, sheep and *Drosophila* [59]. Recently, Zhu et al, (2017) functionally tested conserved candidate genes in *Drosophila* with de novo mutations identified through a genomic association study from a large number of severe cases of CHD [60]. This screen, based on heart-specific RNAi silencing, pinpointed 70 genes essential for the development, structure, or function of the *Drosophila* heart. Interestingly, among the new potential CHD genes, several were found to be involved in histone H3K4 and H3K27 methylation (*Kismet/CHD7*, *WDS/WDR5*, *Trx/MLL2*, *Lid/KDM5A-B*) [60]. The silencing of genes responsible for H3K4 and H3K27 methylation, but not demethylation, induced developmental lethality, severe structural heart abnormalities, and/or reduced lifespan [60]. The function of these genes in CHDs was validated by replacing the endogenous fly genes with the patient-derived mutant alleles, resulting in similar cardiac symptoms reversed by overexpressing a wild-type human gene in the *Drosophila* heart [60]. *Drosophila* thus helped to identify genes involved in histone H3K4 and H3K27 methylation as new CHD genes.

Another recent study [61] focused on de novo copy number variants (CNVs) detected in a cohort of CHD patients. The heart-specific RNAi attenuation of CNV candidate genes led to the identification of *RpL13* (*ribosomal protein L13*), which encodes a subunit of the large cytosolic ribosome complex and *SON*, a splicing cofactor. Intriguingly, heart-specific *RpL13* knockdown at embryonic stages and at the early L1 larval stage resulted in a complete loss of the heart, while *RpL13* attenuation in later larval stages did not lead to any heart defect [61]. Because *RPL13* is involved in proliferation and differentiation of human cardiac progenitors and plays an essential role in fly heart development, it represents a new candidate CHD gene. Similarly, the attenuation of *SON* leads to a reduced fly heart contractility that mimics the cardiac phenotype of CNV patients and suggests a role in CHD [61].

The conservation of genes controlling the early stages of heart development between *Drosophila* and humans thus makes the fruit fly a highly valuable model for testing and identifying new CHD genes. However, *Drosophila* can also be used to model human diseases in which cardiac function is affected. As an example, we discuss below the modeling myotonic dystrophy type 1 characterized by severe heart dysfunction.

### 4.3. Modeling Myotonic Dystrophy Type 1 Heart Defects

Myotonic dystrophy type 1 (DM1, OMIM #160900), an autosomal dominant disorder, is the most prevalent type of muscular dystrophy in adults [62], with an estimated incidence of 1/8000 births [63]. DM1 symptoms include progressive muscle weakness, myotonia, respiratory failure and cardiac dysfunctions. DM1 patients also develop early onset cataracts, testicular atrophy, insulin resistance, and neurological problems [63,64]. However, respiratory failure and heart defects are the main causes of mortality in DM1.

**Figure 2:**
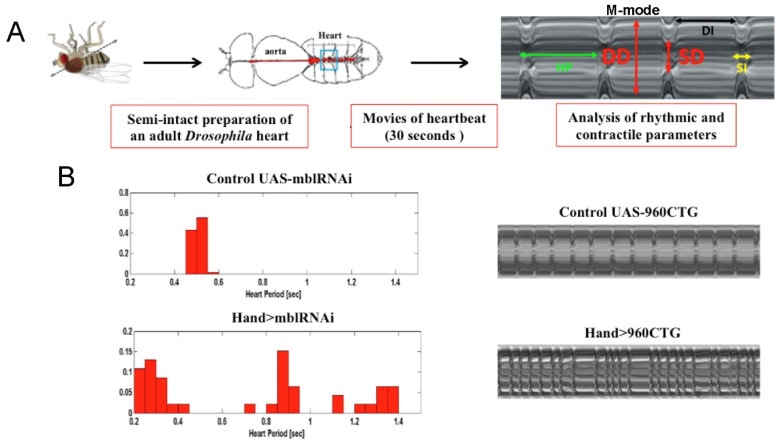
Modeling cardiac DM1 symptoms in *Drosophila*. (**A**) Scheme illustrating semi-intact preparation of adult *Drosophila* heart for semi-automated optical heartbeat analysis (SOHA). A blue window indicates the imaged heart area. On the right, an example of M-mode generated by SOHA shows different rhythmic and contractile heart parameters. HP: heart period; DI: diastolic interval; SI: systolic interval; DD: diastolic diameter; SD: systolic diameter. (**B**) Generated DM1 *Drosophila* lines recapitulate cardiac arrhythmia phenotypes observed in DM1 patients. SOHA analyses of heart-specific attenuation of MBNL1 orthologue (*Hand > mblRNAi*) show high variability of the heart period (lower left panel). M-modes of DM1 fly hearts expressing 960 CTG repeats (*Hand > 960CTG*) show an irregular arrhythmic pattern (lower right panel).

DM1 is caused by the expansion of unstable CTG triplet repeats in the 3′-untranslated region of the *dystrophia myotonica protein kinase* (*DMPK*) gene [65]. The *Dmpk* transcripts carrying expanded CUG triplets (more than 50 and up to several thousand) form toxic hairpin-like secondary structures in the nuclei that sequester RNA-binding proteins (RNBPs), and in particular Muscleblind-like 1 (MBNL1), leading to loss of its function [66,67]. In parallel, another RNBP, ELAV-like factor 1 (CELF1), becomes stabilized [68]. MBNL1 and CELF1 regulate the alternative splicing of common targets involved in skeletal and cardiac muscle function, and their misbalance causes splicing defects responsible for DM1 symptoms [69]. Cardiac dysfunctions appear in 80% of DM1 patients, generally several years after the onset of neuromuscular symptoms, but in some cases, cardiac disease may be the first manifestation in DM1. Among cardiac defects, DM1 patients present an increased risk of mechanical diastolic and/or systolic dysfunction, cardiomyopathy, conduction disturbances and arrhythmias [70], but the most common DM1 cardiac defect is conduction disturbances with lengthened PR interval (corresponding to the time between atrial depolarization and ventricular depolarization) and intraventricular conduction delay [71,72]. To identify gene deregulations caused by toxic repeats and underlying DM1 pathogenesis, several animal models were generated [65,66]. Importantly, *Drosophila* DM1 models [73,74] have reproduced all the cardiac defects observed in DM1 patients, with the analysis of phenotypes facilitated by semi-automated optical heartbeat analysis (SOHA) [75]. Briefly, this technique allows the analysis and quantification of rhythmicity and the dynamics of the heart contractions, including the relaxation phase (DI, diastolic interval), contraction phase (SI, systolic interval), heart period (HP), arrhythmia index (AI), end systolic diameter (ESD), end diastolic diameter (EDD), and the percentage of fractional shortening (Figure 2A).

Recent work by our group [76] revealed that heart-specific DM1 models showed the asynchronous propagation of cardiac contraction waves mimicking the conduction defects observed in DM1 patients. To identify genes involved in DM1-associated conduction disturbances, transcripts were isolated specifically from the DM1 fly hearts using a TU-tagging approach, followed by RNA sequencing [76]. This showed that the expression of *straightjacket (stj)*/*Calcium Voltage-Gated Channel Auxiliary Subunit Alpha 2 delta 3 (CACNA2D3)*/*α2δ3*), involved in heart contraction, was significantly increased in the cardiac cells of DM1 flies [76]. Consistent with this observation, the targeted overexpression of *stj* in the *Drosophila* heart led to conduction defects, and its attenuation in DM1 flies rescued this heart phenotype. The important role of precise regulation of cardiac *stj/α2δ3* expression levels was further supported by an increase in ventricular *α2δ3* expression level in DM1 patients with cardiac conduction defects. Altogether, these findings point to a possible *α2δ3*-targeted drug-based therapy to ameliorate conduction disturbances in DM1 [76].

As stated before, fly models also reproduce other DM1-associated cardiac phenotypes, including arrhythmias, increased heart period, and affected contractility [76,77,78] (Figure 2B). An extensive analysis of heart function was performed in DM1 flies with cardiac expression of 250 CUG repeats [78]. These flies showed severe heart defects, which could all be rescued by overexpressing Muscleblind (Mbl), the *Drosophila* MBNL1 orthologue, thus suggesting that the sequestration of MBNL1 is the main cause of heart dysfunction in DM1.

Based on this observation, a large screen for potential therapeutic compounds that inhibit the binding of MBNL1 to CUG repeats was performed [79]. The potential drugs were tested in vitro using a real-time fluorescence polarization/anisotropy assay that consisted of incubating drugs with fluorescently labeled 250 CUG repeats and with purified recombinant MBNL1. The difference between the fluorescence polarization of 250 CUG free in solution and 250 CUG in a complex with MBNL1 identified entities that interfered with MBNL1 binding to CUG repeats. The final screen of 12 preselected compounds in primary cultures of myoblasts from DM1 patients identified daunorubicin hydrochloride, an RNA intercalant that binds to and stabilizes CUG RNA structure, efficiently limiting MBNL1 binding [79].

Interestingly, when DM1 flies carrying 250 CUG repeats were fed daunorubicin, Mbl was homogeneously distributed in the nuclei of cardiomyocytes, and ribonuclear foci were reduced, confirming the efficiency of this substance in inhibiting the binding of Mbl to CUG repeats [79]. The analysis of the cardiac physiology of DM1 flies showed that daunorubicin treatment led to a greater improvement in heart performance than treatment by pentamidine, another anti-DM1 drug [79]. Both substances were able to rescue arrhythmias and heart contractility, but daunorubicin was more efficient in restoring DM1 systolic and diastolic heart functions [78,79]. These studies thus demonstrate that the fruit fly heart can offer a good in vivo system to dissect heart dysfunctions in DM1 and identify potential therapeutic compounds.

## 5. Conclusions

The early stages of development of both the *Drosophila* and vertebrate heart show remarkable similarities, with conserved genes that control early cardiogenesis. This makes the fly heart a model system well suited to studying cardiogenic pathways, and also to identifying the genes involved in complex and highly heterogenous congenital heart disorders (CHDs).

Despite morphological differences, the fly heart also offers an attractive model to study mechanisms controlling cardiac function and associated disorders. The *Drosophila* model has helped to identify novel genes and pathways involved in arrhythmias [47], atrial fibrillation [80], and channelopathies [81]. It has also been applied to modeling cardiac aging [51], and cardiomyopathies including dilated [82,83,84,85,86,87], hypertrophic [88,89,90], and restrictive cardiomyopathy [82,91,92], in which heart failure could occur [43]. Finally, the developed *Drosophila* models of muscular diseases with cardiac symptoms such as DM1 have been instrumental in dissecting the gene deregulations underlying heart defects and in identifying new therapeutic strategies.

## Data Availability

Not applicable.
